# Super Annigeri 1 and improved JG 74: two Fusarium wilt-resistant introgression lines developed using marker-assisted backcrossing approach in chickpea (*Cicer arietinum* L.)

**DOI:** 10.1007/s11032-018-0908-9

**Published:** 2018-12-28

**Authors:** D. M. Mannur, Anita Babbar, Mahendar Thudi, Murali Mohan Sabbavarapu, Manish Roorkiwal, Sharanabasappa B. Yeri, Vijay Prakash Bansal, S. K. Jayalakshmi, Shailendra Singh Yadav, Abhishek Rathore, Siva K. Chamarthi, Bingi P. Mallikarjuna, Pooran M. Gaur, Rajeev K. Varshney

**Affiliations:** 1 Agricultural Research Station, University of Agricultural Sciences (UAS)-Raichur, Kalaburagi, Karnataka 585 101 India; 2 Jawaharlal Nehru Krishi Vishwa Vidyalaya (JNKVV), Jabalpur, Madhya Pradesh 482 004 India; 3 International Crops Research Institute for the Semi-Arid Tropics (ICRISAT), Patancheru, Telangana 502 324 India

**Keywords:** Chickpea, Fusarium wilt, Foreground selection, Background selection, Marker-assisted backcrossing

## Abstract

Annigeri 1 and JG 74 are elite high yielding desi cultivars of chickpea with medium maturity duration and extensively cultivated in Karnataka and Madhya Pradesh, respectively. Both cultivars, in recent years, have become susceptible to race 4 of Fusarium wilt (FW). To improve Annigeri 1 and JG 74, we introgressed a genomic region conferring resistance against FW race 4 (*foc4*) through marker-assisted backcrossing using WR 315 as the donor parent. For foreground selection, TA59, TA96, TR19 and TA27 markers were used at Agricultural Research Station, Kalaburagi, while GA16 and TA96 markers were used at Jawaharlal Nehru Krishi Vishwa Vidyalaya, Jabalpur. Background selection using simple sequence repreats (SSRs) for the cross Annigeri 1 × WR 315 in BC_1_F_1_ and BC_2_F_1_ lines resulted in 76–87% and 90–95% recurrent parent genome recovery, respectively. On the other hand, 90–97% genome was recovered in BC_3_F_1_ lines in the case of cross JG 74 × WR 315. Multilocation evaluation of 10 BC_2_F_5_ lines derived from Annigeri 1 provided one superior line referred to as Super Annigeri 1 with 8% increase in yield and enhanced disease resistance over Annigeri 1. JG 74315-14, the superior line in JG 74 background, had a yield advantage of 53.5% and 25.6% over the location trial means in Pantnagar and Durgapura locations, respectively, under Initial Varietal Trial of All India Coordinated Research Project on Chickpea. These lines with enhanced resistance and high yield performance are demonstration of successful deployment of molecular breeding to develop superior lines for FW resistance in chickpea.

**Electronic supplementary material:**

The online version of this article (10.1007/s11032-018-0908-9) contains supplementary material, which is available to authorized users.

## Introduction

Chickpea (*Cicer arietinum* L.) is the second most important food legume, cultivated on residual soil moisture by the resource poor farmers in the South Asia, Indian sub-continent and Sub-Saharan Africa. Besides being protein-rich source for human diet, it also improves soil health and structure by adding nitrogen through symbiotic association with Rhizobium. In addition, valuable malic acid is added to soil through massive amount of fallen leaves which increases nutrient availability. In India, it is grown in a wide range of agro-climatic niches. Based on crop duration, these regions are classified as short (Southern/peninsular India), medium (Central India) and long (Northern India) duration environments. The major chickpea growing countries include India (67.4%), Australia (6.21%), Pakistan (5.73%), Turkey (3.86%), Myanmar (3.74%) and Iran (2.25%). Approximately 9.38 m tons of chickpea was produced during 2016–2017 (http://agricoop.gov.in/sites/default/files/2ND_ADV_EST_APY_201718_E.pdf).

In India, the chickpea area dramatically declined from 4.7 to 0.7 m ha in northern states like Punjab, Haryana and Uttar Pradesh between 1965 and 2010 and increased from 2.1 to 6.1 m ha in central and southern states like Madhya Pradesh, Maharashtra, Andhra Pradesh and Karnataka between 1967 and 2012 (Gowda et al. 2013). This major shift in the cultivated area of chickpea is due to (i) green revolution that intensified irrigated wheat cultivation and (ii) availability of short duration chickpea varieties. Despite of increase in area and production, the productivity has been less than one ton per ha mainly due to adverse effects of biotic and abiotic stresses. The emergence of Fusarium wilt (FW) as a devastating root disease of chickpea in central and southern India has been leading to 100% yield losses under favourable conditions (Halila and Strange 1996; Sharma et al. 2004; Jendoubi et al. 2017). *Fusarium oxysporum* f. sp. *ciceris* is a soil-borne pathogenic fungus, which causes FW and differs in pathogenic variability. Based on variation in virulence among isolates of *foc* races, eight distinct physiological races were reported namely races 0, 1A, 1B/C, 2, 3, 4, 5 and 6 (Jendoubi et al. 2017). Among eight races known, the genetics of resistance to six races has been studied extensively (Singh et al. 1987; Kumar 1998; Tullu et al. 1999; Tekeoglu et al. 2000; Rubio et al. 2003; Sharma et al. 2004, 2005). The race 4 is highly prevalent in Karnataka and Madhya Pradesh states. Earlier studies reported that resistance to races 0, 1A, 2 and 4 is either digenic or trigenic, whereas resistance to races 3 and 5 is monogenic (Tullu et al. 1999; Tekeoglu et al. 2000).

Recent advances in genomics research enabled development and application of molecular markers for crop improvement (Thudi et al. 2014; Varshney et al. 2018). In the case of chickpea, ample genomic resources in the form of molecular markers (Nayak et al. 2010; Thudi et al. 2011; Hiremath et al. 2012), genetic maps (Nayak et al. 2010; Thudi et al. 2011; Varshney et al. 2014a), physical map (Varshney et al. 2014b), the draft genome sequence of kabuli chickpea genotype CDC Frontier (Varshney et al. 2013a) and resequencing of several germplasm lines (Thudi et al. 2016a, b) have now become available for chickpea improvement. The genes/QTLs for resistance to six races (0, 1A, 2, 3, 4 and 5) of FW pathogen have been mapped on to the chickpea genetic map (Sharma et al. 2005; Sabbavarapu et al. 2013; Li et al. 2015). Some superior lines with enhanced tolerance or resistance to abiotic and biotic stresses as well as economically important traits have been developed in legumes (Lucas et al. 2015; Varshney 2016; Parhe et al. 2017; Varshney et al. 2018) using marker-assisted backcrossing (MABC). In the case of chickpea, a genomic region (known as “*QTL-hotspot*”) harbouring several QTLs for drought component traits was identified (Varshney et al. 2014a) and successfully introgressed initially into an elite cultivar JG 11 (Varshney et al. 2013b). Preliminary yield trials indicated 12–24% increase in yield under drought conditions. In addition, the introgression of this genomic region into different genetic backgrounds like KAK 2 and Chefe was also found to enhance the drought tolerance. Further, this genomic region is being intogressed into elite cultivars in Kenya, Ethiopia and India (Thudi et al. 2017). The results encouraged us to harness these resources and enhance FW resistance in elite chickpea cultivars of Karnataka and Madhya Pradesh.

In a recent base line survey, high yielding, drought tolerance and FW resistance were most preferred characters by farmers and farm women in Karnataka and Madhya Pradesh with highest Garrett Scores (http://www.icrisat.org/what-we-do/impi/projects/tl2-publications/research-reports/rr-cpkrn.pdf; Ghosh et al. 2014) with an intense emphasis on chickpea cultivars with increased FW resistance. Hence, there is a need to develop high yielding chickpea cultivars with FW resistance. In the present study, we report development of Super Annigeri 1 and improved JG 74 with enhanced yield and resistance to FW using MABC approach.

## Materials and methods

### Plant material

Annigeri 1 and JG 74 suceptible to FW race 4 (*foc4*) were used as recipient parents and WR 315; a chickpea landrace resistant to FW race 4 (*foc4*) was used as donor parent. Annigeri 1 was developed and released for cultivation, from a landrace Annigeri, in 1978 by University of Agricultural Sciences (UAS), Bangalore. Annigeri 1 is semi-spreading cultivar with medium seed size (16–20 g /100 seeds), matures in 95–100 days and has an average yield of 9–12 Q/ha (Bantilan et al. 2014). JG 74 is an early maturing variety (110–115 days duration) released in 1991 by JNKVV, Jabalpur is most suitable for rainfed conditions of Madhya Pradesh and it has an average yield of 11–13 Q/ha (http://farmer.gov.in/imagedefault/pestanddiseasescrops/pulses.pdf).

### DNA extraction and marker genotyping

Young leaf tissues were collected from 20-day-old seedlings and genomic DNA was extracted by following high-throughput mini-DNA extraction protocol as described by Cuc et al. (2008). The DNA was checked for its quantity and quality on 0.8% agarose gel.

Depending on marker polymorphism between donor and recipient parents, 2–4 simple sequence repeat (SSR) markers (TA59, TA96, TR19, TA27) linked to FW resistance QTLs on CaLG02 and an adjacent marker GA16 were used for foreground selection (Sharma and Muehlbauer 2005; Millan et al. 2006) (Table S1). For background selection, SSR markers equally distributed on chickpea genome were used (Table S2). PCR of SSR markers used for foreground selection and background selection, visualisation of amplified products, separation by capillary electrophoresis was performed as described in the earlier studies (Nayak et al. 2010; Thudi et al. 2011).

Recurrent parent genome recovery (RPGR) is calculated as per Sundaram et al. (2008). In brief, recurrent parent genome recovery

$$ G=\left[\left(X+0.5\mathrm{Y}\right)\times 100\right]\ N $$

where

*N*: total number of parental polymorphic markers screened.
*X*: number of markers showing homozygosity for recurrent parent allele.
*Y*: number of markers showing heterozygosity for parental alleles.

Graphical genotypes were drawn using GGT v.2.0 (van Berloo 2008).

### Crossing and selection of genotypes

#### MABC at ARS-Kalaburagi

At ARS-Kalaburagi, Annigeri 1 × WR 315 crosses were made using Annigeri 1 as recipient and WR 315 as donor in the crop season of 2009–2010. F_1_ seeds were harvested and sown in the next crop season of 2010–2011. In this season, the confirmed hybrids (F_1_s), after verifying with the help of molecular markers, were used as pollen parent to make first backcross with Annigeri 1. In the BC_1_F_1_ and BC_2_F_1_ generations in subsequent crop seasons (2011–2012 and 2012–2013), individual plants that were heterozygous at the *foc4* locus based on foreground selection and having higher recurrent parent genome recovery (RPGR) based on background selection were identified and used for subsequent crossing. In parallel, the desirable plants in BC_2_F_1_ were selfed to obtain BC_2_F_2_ in the crop season of 2012–2013.

#### MABC at JNKVV, Jabalpur

At JNKVV, Jabalpur, JG 74 × WR 315 crosses were made using JG 74 as recipient and WR 315 as donor in the crop season of 2009–2010. F_1_ seeds were harvested and sown in the next crop season of 2010–2011. In this season, the confirmed hybrids (F_1_s), after verifying with the help of molecular markers, were used as pollen parent to make first backcross with JG 74. In the BC_1_F_1_ (crop season, 2010–2011) and BC_2_F_1_ (off-season, 2011), individual plants that were heterozygous at the *foc4* locus based on foreground selection were identified and used for subsequent crossing. Recurrent parent genome recovery was estimated in BC_3_F_1_ generation.

### Phenotyping of MABC lines for FW resistance

#### Agricultural Research Station-Kalaburagi

The MABC lines (BC_2_F_2_ progenies) along with the parental lines and susceptible check (JG 62) were phenotyped for resistance to race 4 of FW in a wilt sick plot at ARS-Kalaburagi, Karnataka, India, during crop season of 2013–2014. The wilt sick plot had sufficient inoculum load as indicated by 100% mortality of the susceptible check JG 62 in the wilt sick plots. Visual observations on appearence of wilt symptoms were recorded at 60 days after sowing and the progenies were classified as resistant (0–20%), moderately resistant (21–50%) and susceptible (> 50%) (Sharma et al. 2005). The superior lines identified in BC_2_F_3_ progenies were evaluated under replicated trials for disease resistance and yield during 2015–2016. The superior lines were identified based on high fusarium wilt resistance and were further evaluated under multi-location testing (MLT) for yield at three locations (Kalaburagi, Bidar and Dharwad) during the crop season 2016–2017.

#### JNKVV, Jabalpur

A total 40 BC_3_F_4_ lines were evaluated at Seed Breeding Farm for agronomic traits and also screened in wilt sick plot at JNKVV, Jabalpur during crop season of 2014–2015. The lines possessing similar agronomic traits like recurrent (JG 74) and resistant to FW were selected. One superior line JG 74315-14 along with local check, recurrent parent and 10 other entries was evaluated in four locations (Jabalpur, Rewa, Ganjbasoda and Sagar) for yield performance and disease reaction under state varietal trials using RBD design during crop season 2016–2017. Disease resistance was scored on 1–10 scale, as described earlier by Varshney et al. (2014c).

### Statsistical analysis

Pooled and location wise analysis of variance was carried out using PROC MIXED (SAS v9.4, SAS Institute Inc. 2017), considering environment, genotype and replication as fixed. In order to pool the data across the environments, individual environment variances were modelled to error distribution using residual maximum likelihood (REML) procedure. Combined and environment wise least square means (LSMeans) were calculated for genotypes and also performed least significant difference (LSD) for significant genotype effects.

## Results

### Markers for foreground and background selection

Based on parental polymorphism, TA82, TA96 and TA27 markers and TA96 and GA16 markers were used at ARS-Kalaburagi and JNKVV, Jabalpur, respectively, for foreground selection (Table S1). A set of 38 and 35 SSR markers distributed uniformly across the chickpea genome were used for background selection in BC_1_F_1_ and BC_2_F_1_ generations, respectively, at ARS-Kalaburagi (Table S2). While a total of 42 polymorphic SSR markers were used for background selection in BC_3_F_1_ at JNKVV, Jabalpur (Table S2)_._

### Introgression of race-specific resistance into Annigeri 1

**Fig. 1:**
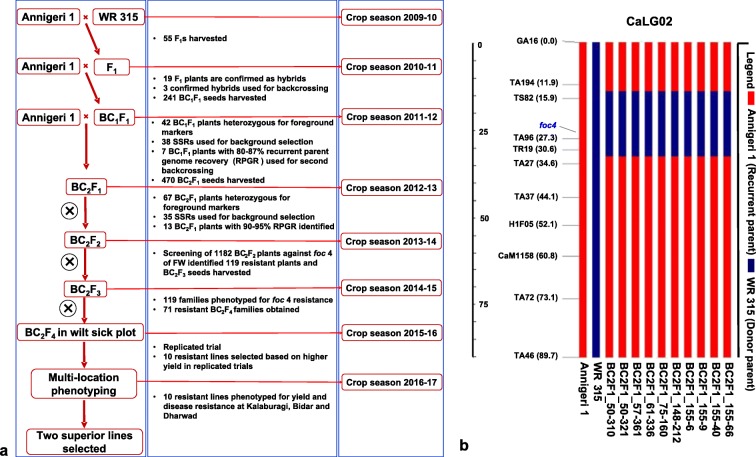
Marker-assisted backcrossing (MABC) scheme and graphical genotype of superior lines**. a** MABC scheme adopted to develop superior lines with enhanced resistance to race 4 of *Fusarium oxysporum* f. sp. *ciceris* in the genetic background of Annigeri 1 using WR 315 as donor. **b** Graphical genotype of 10 BC_2_F_1_ lines for CaLG02. A total of 38 and 35 SSRs equally distributed on the genome were used for background selection in BC_1_F_1_ and BC_2_F_1_ generations, respectively. Three SSR markers (TA96, TS82 and TR19) and eight SSR markers (GA16, TA194, TA27, TA37, H1F05, CaM1158, TA72 and TA46) were used selecting the donor alleles and recurrent parent genome recovery on CaLG02

A total of 55 F_1_s were harvested from the cross Annigeri 1 × WR 315 at ARS, Kalaburagi during crop season 2009–2010 (Fig. 1a). These F_1_s were planted during the crop season of 2010–2011 and of the 55 F_1_s, 19 plants were confirmed as hybrids using polymorphic markers (TA194, TS82 and GA16). Out of 19 confirmed F_1_s, only three F_1_s were used to make first backcross with Annigeri 1 and generated 241 BC_1_F_1_ seeds. A total of 188 plants obtained from 241 BC_1_F_1_ seeds were used for marker analysis (foreground selection) during the crop season of 2011–2012. As a result, 42 plants were found heterozygous for three markers (TS82, TA96 and TR19). These 42 plants were further subjected to background selection with 38 SSR markers (Table S2) and identified recurrent parent genome recovery (RPGR) in the range of 76–87% (Table S3). Among these, seven plants with highest RPGR (80.8–87%) were used for second cycle of backcrossing, and as a result, 470 BC_2_F_1_ seeds were harvested. During the crop season 2012–2013, a total of 376 plants obtained from 470 BC_2_F_1_ seeds were used for marker analysis (foreground selection). As a result, 64 plants were identified as heterozygous for all three foreground markers. On assessing the RPGR of 64 plants using 35 SSR markers (Table S2), 18 plants with high RPGR (90–95%) were selected (Table S4). A total of 1182 BC_2_F_2_ seeds were harvested from 13 BC_2_F_1_ plants. During crop season 2013–2014, out of the 1182 seeds sown in wilt sick plot, a total of 935 seedlings germinated and on subsequent wilt screening, 816 plants were wilted and a total of 119 plants were identified as resistant to race 4 of FW. A total of 9157 BC_2_F_3_ seeds were harvested from 119 resistant plants (Table S5). The graphical genotype of targeted introgressed donor segment and percentage of background genome recovered in 10 BC_2_F_1_ lines on CaLG02 is summarised in Fig. 1b.

### Introgression of race-specific resistance into JG 74

**Fig. 2:**
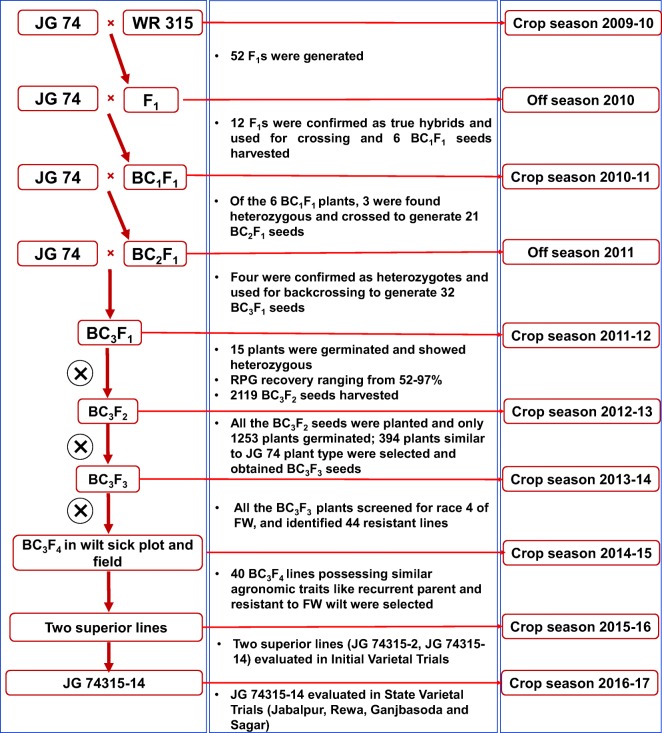
Marker-assisted backcrossing (MABC) scheme to develop superior lines with enhanced resistance to race 4 of *Fusarium oxysporum* f. sp. *ciceris* in the genetic background of JG 74 using WR 315 as donor**.** Two SSR markers (GA16 and TA96) were used for foreground selection, 42 SSRs were used for background selection in BC_3_F_1_

By using JG 74 as recipient and WR 315 as donor, the cross JG 74 × WR 315 was made during crop season 2009–2010 to generate F_1_s. Out of 52 F_1_ plants generated, 12 F_1_s were confirmed as hybrids using the polymorphic markers (TA96 and GA16) during off-season (July 2010) (Fig. 2). All F_1_s were used to make first backcross [(JG 74 × (JG 74 × WR 315)]; however, only six BC_1_F_1_ seeds were harvested in September, 2010. All the six BC_1_F_1_ seeds were sown in the crop season 2010–2011. Foreground selection was done with two SSR markers namely GA16 and TA96. As a result, three heterozygous plants identified were used for second cycle of backcrossing and 21 BC_2_F_1_ seeds were harvested in April, 2011 (Table S6). These 21 BC_2_F_1_ seeds were sown in the off-season (August–November, 2011). After foreground marker analysis, four BC_2_F_1_ heterozygous plants were used for third backcrossing. As a result, 32 BC_3_F_1_ seeds were obtained in November 2011. Although all 32 BC_3_F_1_ seeds were sown in the crop season December 2011–2012, only 15 BC_3_F_1_ plants were obtained. All 15 plants were found heterozygotes. Background selection based on 42 SSR markers (Table S2) showed 52–97% RPGR in these plants. A total of 2119 BC_3_F_2_ seeds were harvested from all these 15 BC_3_F_1_ plants in April 2012 (Table S7) and a total of 394 BC_3_F_2_ plants were selected based on JG 74 plant type. From 394 BC_3_F_2_ plants, a total of 66,076 BC_3_F_3_ seeds were obtained. Approximately 10,000 BC_3_F_4_ seeds were harvested from 44 BC_3_F_3_ lines during crop season 2013–2014.

### Phenotypic performance of Annigeri 1 MABC lines

#### Disease resistance in wilt sick plot

During 2013–2014 crop season, 9157 seeds from 119 resistant BC_2_F_2_ plants were grown as plant to row progenies in wilt sick plot along with the parental lines and wilt susceptible check (JG 62) at ARS-Kalaburagi, Karnataka. Wilt screening revealed that among 119 BC_2_F_3_ progenies, 71 families were found uniformly resistant to FW, whereas remaining 48 families exhibited either susceptible reaction or segregated into susceptible and resistant types. These 71 resistant lines were advanced to test in replicated trial for yield during the crop season 2014–2015 and 2015–2016. On evaluation of 71 resistant lines under replicated trials during 2015–2016, 10 best lines with high resistance to FW (disease score ranging from 0 to 4.35%) and seed yield (ranging from 1366.67 to 1894.79 kg/ha) were identified (Table S8). The 10 superior lines having high FW resistance were named as Super Annigeri 1 (SA-1) lines (SA1-1, SA1-2, SA1 -3, SA1-4, SA1-5, SA1-6, SA1-7, SA1-8, SA1-9 and SA1-10). These lines were further evaluated for yield in three locations during the crop season 2016–2017.

#### Multi-location evaluation of superior lines of chickpea

**Fig. 3:**
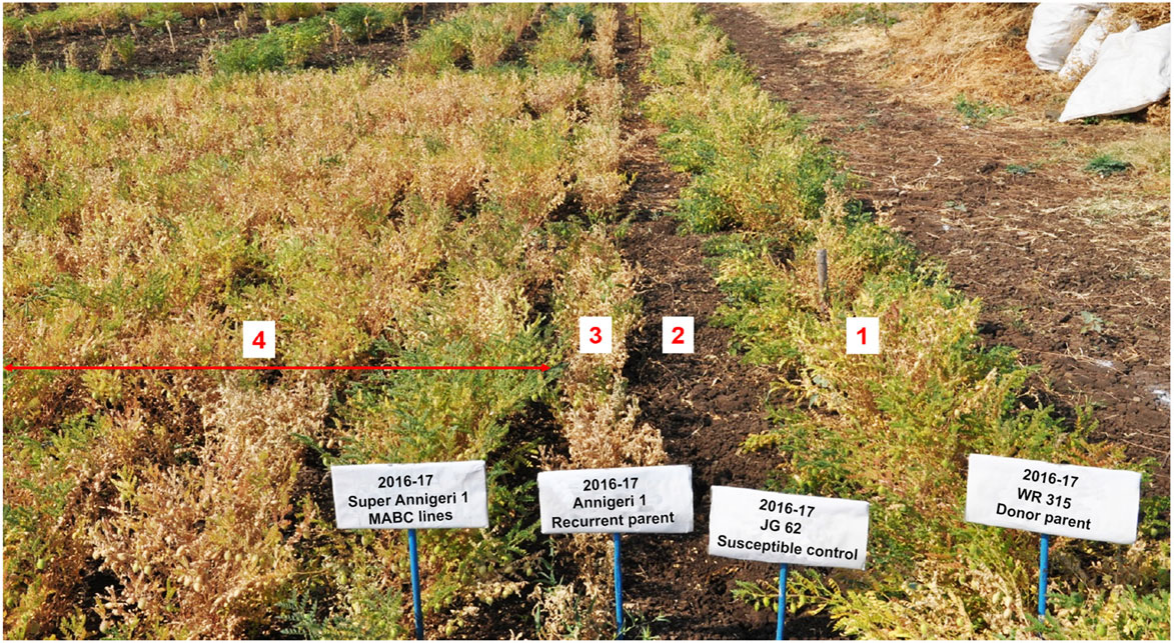
Evaluation of Super Annigeri lines with Annigeri 1 (recurrent parent), JG 62 (susceptible check) and WR 315 (donor parent) in wilt sick plot at ARS, Kalaburagi during 2016–2017. WR 315, the donor for FW (see row numbered 1); JG 62, the susceptible check, completely wilted and the plants are uprooted (see row numbered 2); wilting symptoms can be seen in case of recipient parent (see row numbered 3); Super Annigeri 1–MABC line with enhanced resistance (see row numbered 3)

Multi-location evaluation of 10 superior lines of Super Annigeri1 (SA-1) were conducted at three locations (Kalaburagi, Bidar and Dharwad) of Karnataka during 2016–2017. The mean yield data from three locations indicated that the line MLT-SA1-1 recorded high yield (1920 kg/ha) followed by MLT-SA1-2 (1835 kg/ha) compared to the check Annigeri 1 (1771 kg/ha) (Table 1). The disease screening of these lines indicated significantly less FW incidence in the case of MLT-SA1-1 (7.10%) and MLT-SA1-2 (7.55%) compared to recurrent parent Annigeri 1 (41.40%) and susceptible check JG 62 (100%) (Fig. 3). Mean yield performance of all MABC lines tested in the genetic background of Annigeri 1 differed significantly (*p* value < 0.05) among themselves at all three locations (Table S9). Among 10 lines, yield performance of MLT-SA1-1 was significantly higher compared to WR-315 (donor parent) in pooled analysis and individual locations except in Dharwad. Further, MLT-SA1-1 also performed better than Annigeri 1 (recurrent parent) in Bidar and JG 11 (local check) in pooled analysis and at Gulbarga (Table S10). In addition, the mean yield performance of MLT-SA1-2 was significantly high compared to WR 315 (donor parent) in all locations. The yield performance of MLT-SA1-2 was also significantly different from Annigeri 1 (recurrent parent) in Bidar and JG 11 (local check) in pooled analysis and at Gulbarga location (Table S11).

**Table 1:** Yield performance and disease reaction of Super Annigeri (SA) lines of chickpea at three locations of Karnataka during 2016–2017

Entries	Yield in kg/ha^*^				Increase in yield over Annigeri 1 (%)	Disease reaction (%)
	Kalaburagi	Bidar	Dharwad	Mean		
MLT-SA1-1	2769	1781	1210	1920	8.4	7.10 (R)
MLT-SA1-2	2718	1812	975	1835	3.6	7.55 (R)
MLT-SA1-3	2606	1674	1023	1768	–	9.01 (R)
MLT-SA1-4	2341	1792	1158	1764	–	12.60 (MR)
MLT-SA1-5	1715	1677	1147	1513	–	22.36 (MS)
MLT-SA1-6	2432	1410	944	1595	–	11.06 (MR)
MLT-SA1-7	2411	1597	1087	1698	–	12.87 (MR)
MLT-SA1-8	2761	1667	1259	1896	6.95	11.05 (MR)
MLT-SA1-9	2665	1524	1323	1837	3.4	12.12 (MR)
MLT-SA1-10	2485	1729	1208	1807	1.9	16.07 (MR)
Annigeri 1 (Recurrent parent)	2684	1444	1186	1771	–	41.40 (S)
JG 11 (Local check)	2115	1715	1177	1669	–	18.77 (MR)
WR 315 (Donor parent)	1941	1385	1348	1558	–	100.00 (HS)
CV at 5%				12.93	–	1.34 (R)
CD (0.05)				379.28	**–**	

*R* resistant, *MR* moderately resistant, *MS* moderately susceptible, *S* susceptible, *HS* highly susceptible; “−” either no increase in yield or not applicable

^*^Statistical significance of yield performance of Super Annigeri lines are provided in Tables S7, S8 and S9

### Phenotypic performance of JG 74 MABC lines

All selected BC_3_F_4_ plants (~ 10,000) were grown and subjected to phenotyping against race 4 of FW under field condition at JNKVV, Jabalpur in the crop season 2013–2014. Transgressive segregation for resistance observed in BC_3_F_4_ (appearance of extreme individuals than the resistant parent) suggests that the parent JG 74 is susceptible to FW and WR 315 possess positive resistant alleles for race 4. The transgressive segregation also suggests the resistance to be quantitative in nature. Two entries (JG 74315-2 and JG 74315-14) based on their performance were nominated for Initial Varietal Trails (IVT) of All India Coordinated Project (AICRP), on chickpea. In IVT trials (crop season 2015–2016), JG 74315-14 was ranked 4th, as it had yield advantage of 53.5% and 25.6% in Pantnagar and Durgapura over the location trial means (Supplementary Information 1). Evaluation for yield performance of JG 74315-14 in state varietal trials in four locations (Jabalpur, Rewa, Ganjbasoda and Sagar) in Madhya Pradesh has shown 125% and 25% mean increase in yield over the JG 74 (recurrent parent) and JG 14 (local check), respectively. The mean disease score of JG 74315-14 was 2, which is highly resistant (Table 2). The yield of JG 74315-14 differed significantly over recurrent parent, local check as well as other MABC lines tested in four locations of Madhya Pradesh in state varietal trials during 2016–2017 (Tables S12 and S13).

**Table 2:** Yield performance and disease reaction of JG 74315-14 at four locations of Madhya Pradesh during 2016–2017 in state varietal trial

Entries	Yield in kg/ha^*^					Increase in yield over JG 74 (%)	Disease reaction (mean)
	Jabalpur	Rewa	Ganjbasoda	Sagar	Mean		
JG 74 × JG 11551	2587.2	2435.1	2099.3	2157.6	2319.8		2.25
JG 12 × JG 16-3	2587.3	2153.4	2467.6	2386.1	2398.6		2.25
JG 2016-45	2104.1	1885.4	2030.9	1843.8	1966.1		3
JG 2016-43	2053.8	1995.8	1788.7	2012.5	1962.7		3.5
JG 2016-44	2096.2	1850.5	2107.3	1789.6	1960.9		3.25
JG 24	2560.3	2399.8	2450.6	2245.4	2414		2
JG 2016-9605	2534.1	2222.8	2187.1	2489.5	2358.4		2
ICC 96029 × JG11551	2877.9	2228.4	2455.5	2951.4	2628.3		2
JG 74315-14	2631.9	2399.2	2523.2	2644.4	2549.7	125	2
JG 12 × JG 16	2230.5	2124.4	2155.9	2517.4	2257.1		2
JG 14 × JG 226	2578.6	2369.7	2249.6	2156.3	2338.6		2.5
JG 14 (local check)	2286.7	1750.3	2006.4	2111.1	2038.6		3.25
JG 74 (recurrent parent)	1130.4	1258.2	1169.2	955.2	1128.3		8
CV (%)	7.63	7.36	6.45	8.25			
CD at 5%	299.22	258.30	231.42	302.37			

^*^Statistical significance of yield performance of JG 74315-14 is provided in Table S10 and Table S11

## Discussion

Fusarium wilt is a major disease of chickpea under dry and warm humid conditions leading to significant yield losses. In recent times, this disease has become a major threat due to changing climatic conditions and also a large shift in chickpea growing area from cool long season environments (Northern India) to warm short season environments (Central and Southern India) which exposes the crop to the disease (Gowda et al. 2013). Since it is a soil-borne disease, chemical control of this pathogen is ineffective. Use of disease-resistant cultivars is the most effective and environmentally safe approach for efficient control of the pathogen (Sharma and Ghosh 2016). In the past, several chickpea improved lines or varieties were developed and released for cultivation in South Asia and Sub-Saharan Africa (see Thudi et al. 2016a). However, the FW-resistant varieties released to date are based on conventional screening of the improved or breeding lines in wilt sick plots. Nevertheless, some of these varieties were reported to be susceptible over a period of time that may be due to variability in wilt incidence and genetic differences among the genotypes, genotype × environment interactions (Neupane et al. 2007; Sharma et al. 2012; Nagar 2013).

Earlier studies revealed that resistance to FW is either monogenic or oligogenic based on the race and the cultivars used in the study. However, resistance to race 4 was reported as recessive and digenic in nature (Tullu et al. 1999). Several studies involving inter- and intraspecific RIL populations revealed the organisation of resistance genes for fusarium wilt races 1, 3, 4 and 5 (*foc*1 and *foc*3, *foc*4 and *foc*5; Mayer et al. 1997; Ratnaparkhe et al. 1998; Tullu et al. 1998; Winter et al. 2000; Sharma et al. 2004) in two adjacent resistance gene clusters on CaLG02 flanked by STMS markers GA16 and TA96 (*foc1*–*foc4* cluster) and TA96 and TA27 (*foc3*–*foc5* cluster), respectively (Millan et al. 2006). Moreover, several potential resistance and pathogenesis-related genes were localised on the CaLG02 (Huettel et al. 2002; Pfaff and Kahl 2003). Millan et al. (2006) therefore speculated that CaLG02 is a hot spot for pathogen defence. Recently, QTLs for *foc4* locus for race 4 were identified by Sharma and Muehlbauer (2005) and Millan et al. (2006). QTLs for resistance to FW were reported earlier mainly on CaLG02 and CaLG04 (Cobos et al. 2009; Gowda et al. 2009). Nevertheless, novel QTLs were also mapped on CaLG05 and CaLG06 (Sabbavarapu et al. 2013; Garg et al. 2018). Previous efforts in identifying different FW-resistant genes (Sharma et al. 2005), race-specific unique and rare alleles (Sharma et al. 2014), tagging of molecular markers linked to resistance (Sharma and Muehlbauer 2005) can accelerate the development of improved cultivars in chickpea through MABC.

Annigeri 1 and JG 74 the most popular varieties in Karnataka and Madhya Pradesh, respectively, have become susceptible to Fusarium wilt due to incidence of the pathogen in farmer fields after 2008–2009. As a result, there has been a significant reduction in breeder seed production and indent for these varieties as per Department of Agriculture and Cooperation, Ministry of Agriculture and Farmers’ Welfare, Government of India (Fig. S1). In view of this, the present study was undertaken to introgress the *foc4* locus conferring resistance to race 4 of FW (prevalent in Karnataka and Madhya Pradesh) into Annigeri 1 and JG 74 by employing MABC at ARS-Kalaburagi and JNKVV, Jabalpur. WR 315, a desi landrace from central India resistant to races like 1A, race 2, race 3, race 4 and race 5 with target loci for resistance to *foc4* (Sharma and Muehlbauer 2005), was chosen as the donor parent.

The marker TA96 has been reported to be linked with resistant genes against all the four prevalent races of wilt viz.: *foc1, foc2, foc3* and *foc4* by the genetic distance of 4.9 cM, 1.5 cM 0.5 cM and 3.3 cM, respectively (Winter et al. 2000; Sharma and Muehlbauer 2007; Cobos et al. 2009; Gowda et al. 2009; Halila et al. 2009). Recombinants were selected after each backcross generation using tightly linked marker TA96 (3.3 cM from the *foc4* locus) and flanking markers TS82, and TR19 in the case of backcross progenies derived from Annigeri 1 × WR 315 cross. While in the case of backcross progenies derived from JG 74 × WR 315 cross, recombinants were selected using tightly linked marker TA96 and flanking markers GA16 and TS82. All markers (GA16, TS82, TA96 and TR19) having the sequence information (primer sequence) were anchored on to the reference genome (Varshney et al. 2013a) on pseudomolecule Ca2. Based on the physical position of the markers, 997 genes were identified in the chickpea genome in the region between GA16 and TR19 markers. Interestingly the Ca_14301, that encodes NB-ARC domain disease resistance protein, closer to TA96 and TR19 linked with *foc4* locus was proven to be important candidate gene for resistance to fusarium disease caused by *foc4* in cucumber (https://patents.google.com/patent/WO2015143867A1/en).

MABC approach helped us to introgress resistance to race 4 (*foc4*) of Fusarium wilt into Annigeri 1 and JG 74 along with rapid RPGR. In the present study, 95% and 97% RPGR was achieved in BC_2_F_1_ and BC_3_F_1_ generations in the case of MABC lines developed in the genetic backgrounds of Annigeri 1 and JG 74, respectively. Similar genome recovery was reported earlier by Varshney et al. (2014c). The use of MABC enabled to achieve 95% RPGR in early generations (BC_2_F_1_ derived from Annigeri 1 × WR 315 cross), which could be otherwise achieved only in BC_3_F_1_ and BC_4_F_1_ through conventional breeding method. Successful application of MABC approach in chickpea in introgressing drought (Varshney et al. 2013b), FW (Varshney et al. 2014c; Pratap et al. 2017), Ascochyta blight (Varshney et al. 2014c) into elite cultivars has unequivocally proved the advantage of MABC in breeding improved cultivars. In chickpea, superior lines with enhanced resistance to *foc1* (Varshney et al. 2014c) and *foc2* (Pratap et al. 2017) were developed in the genetic backgrounds of C 214 and Pusa 256 elite chickpea cultivars, respectively. Hence, MABC can be considered as fast-track method to breed and develop the resistant varieties for biotic stresses like FW. This has also clearly indicated the advantage of using codominant markers like SSRs for background selection in recovering higher per cent of RPGR in early generations. Higher RPGR was reported using as low as 13 markers in groundnut (Varshney et al. 2014d) to as high as 88 markers in rice (Vu et al. 2012). Recent studies revealed that MABC involving two improved or released varieties can further reduce the time period for improving popular varieties (Iftekharuddaula et al. 2011; Septiningsih et al. 2015).

Field level phenotyping of backcross progenies was carried out to confirm the wilt reaction. During wilt screening, 10 backcross progenies with highest level of RPGR exhibited a high level of resistance (0–4.35%) compared to the disease incidence in Annigeri 1 (70%) and susceptible check JG 62 (100%). Evaluation of these 10 superior lines for yield performance across 3 locations of Karnataka provided 2 superior lines, i.e. MLT-SA1-1 (1920 kg/ha) and MLT-SA1-2 (1835 kg/ha) with the highest mean yield 8% and 3.6% increase over Annigeri 1 (1771 kg/ha), respectively (Table 1). Also, these improved lines showed good level of FW resistance, while the recurrent parent Annigeri 1 was observed as susceptible. The yield performance of MLT-SA1-1 and MLT-SA1-2 differed significantly (*p* value < 0.05) over the recurrent and donor parents as well as local check at Bidar and Kalaburagi during 2016–2017. These two superior MABC lines were proposed to evaluate in Advanced Varietal Trials (AVT) under All India Coordinated Research Project (AICRP) on chickpea to assess their performance at national level for possible release as improved cultivars for commercial cultivation. At JNKVV, Jabalpur after generating F_1_s, and three rounds of backcrossing (BC_1_F_1_, BC_2_F_1_, BC_3_F_1_) and selfing (BC_3_F_2_, BC_3_F_3_, BC_3_F_4_) the backcross progenies were phenotyped in wilt-sick plot and selected only the positive plants with high level of resistance from each generation against race 4 (*foc4*). In state varietal trials, mean disease reaction of JG 74315-14 was 2, which is highly resistant (Fig. S2). In terms of yield performance, JG 74315-14 has 125% and 25% mean increase in yield over the JG 74 (recurrent parent) and JG 14 (local check), respectively (Table 2). The yield of JG 74315-14 differed significantly over recurrent parent, local check as well as other MABC lines tested in four locations of Madhya Pradesh in state varietal trials during 2016–2017.

In summary, using MABC approach, we successfully introgressed resistance to FW race 4 (*foc4*) into Annigeri 1 and JG 74, the ruling varieties in Karnataka and Madhya Pradesh, respectively, which became susceptible to FW. We also demonstrated 125% and 25% mean yield advantage of JG 74315-14 over recurrent parent and local check, respectively, in the state varietal trials conducted during 2016–2017. While Super Annigeri 1 has 8% mean yield advantage over the Annigeri 1, the improved version of Annigeri 1 based on screening in Kalaburagi, Bidar and Dharwad during 2016–2017. These improved lines with enhanced resistance can be released as varieties for cultivation and also can be used as donors for enhancing disease resistance in other elite cultivars.

## Electronic supplementary material

Fig. S1 Trends in National indent and actual production of breeders seed of popular varieties (a) Annigeri 1 (b) JG 74 from 2008–2009 to 2016–2017 as per Department of Agriculture and Cooperation, Ministry of Agriculture and Farmers’ Welfare, Government of India (PDF 10 kb)

Fig. S2 Evaluation of JG 74315-14 with JG 74 (recurrent parent) and donor WR 315 in wilt sick plot at JNKVV, Jabalpur during 2016–2017. (a) Wilting symptoms can be seen clearly in JG 74, the recurrent parent and (b) while no wilting WR 315, the donor for FW (c) JG 74315-14–MABC line with enhanced resistance (JPG 4545 kb)

Table S1 Details of foreground markers and their physical positions on chickpea genome (XLSX 12 kb)

Table S2 Genetic position of SSR markers used for foreground and background selection of MABC lines in Annigeri 1 and JG 74 genetic backgrounds (DOCX 17 kb)

Table S3 Summary of MABC activities for introgressing resistance to race 4 (*foc*4) into Annigeri 1 variety at ARS-Kalaburagi (DOCX 14 kb)

Table S4 Percent recurrent parent genome recovery in BC_2_F_1_ progenies of cross Annigeri 1 × WR 315 (DOCX 14 kb)

Table S5 Details of BC_2_F_3_ seeds harvested from at ARS-Kalaburagi during crop season 2013–14 (DOCX 14 kb)

Table S6 Summary of MABC activities for introgressing resistance to race 4 (*foc4*) in JG 74 variety using JG 74 × WR 315 cross at JNKVV, Jabalpur (DOCX 14 kb)

Table S7 Details of BC_3_F_2_ seeds in the genetic background of JG 74 and germination status in the field (DOCX 15 kb)

Table S8 Yield performance and disease reaction of 10 best lines in wilt sick plot at ARS-Kalaburagi during 2015–2016 (DOCX 14 kb)

Table S9 Analysis of variance for yield performance of Super Annigeri lines during 2016–2017 (DOCX 12 kb)

Table S10 Yield performance of Super Annigeri line (MLT-SA-1) over recurrent parent, donor and local check during 2016–2017 (DOCX 12 kb)

Table S11 Yield performance of Super Annigeri line (MLT-SA1-2) over recurrent parent, donor and local check during 2016–2017 (DOCX 13 kb)

Table S12 ANOVA for yield performance of JG 74315-14 at four locations of Madhya Pradesh in state varietal trial during 2016–2017 (DOCX 12 kb)

Table S13 Analysis of variance for yield performance of JG 74315-14 over recurrent parent and local check during 2016–2017 (DOCX 12 kb)

ᅟ Supplementary Information 1 Yield performance of JG 74315-14, a superior line in the genetic background of JG 74 in IVT trials during 2016–2017 (DOCX 763 kb)
