# Supplementary figures and images for: Super Annigeri 1 and improved JG 74: two Fusarium wilt-resistant introgression lines developed using marker-assisted backcrossing approach in chickpea (Cicer arietinum L.)
Source: Mol Breed. 2018 Dec 28;39(1):2. doi: 10.1007/s11032-018-0908-9 (PMC6308216; doi:10.1007/s11032-018-0908-9)

**a**

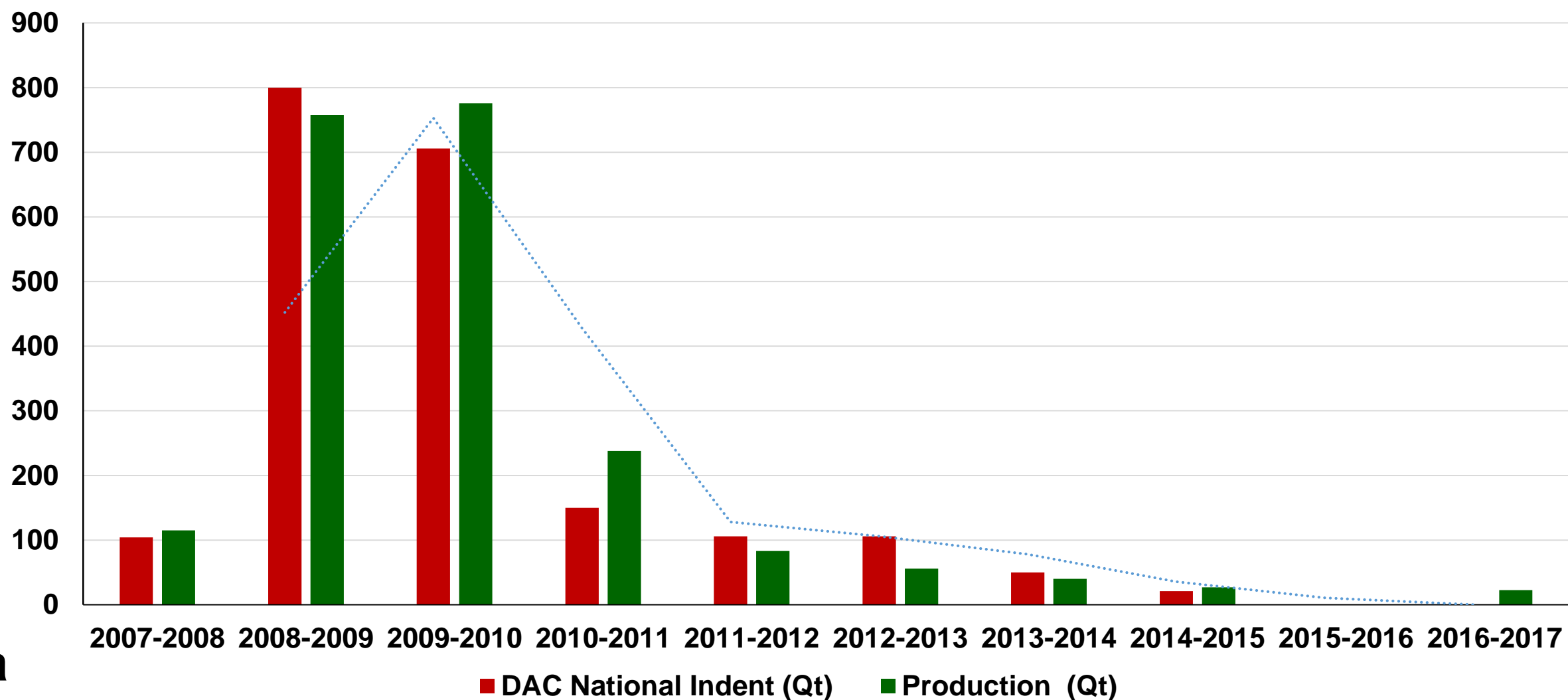

**b**

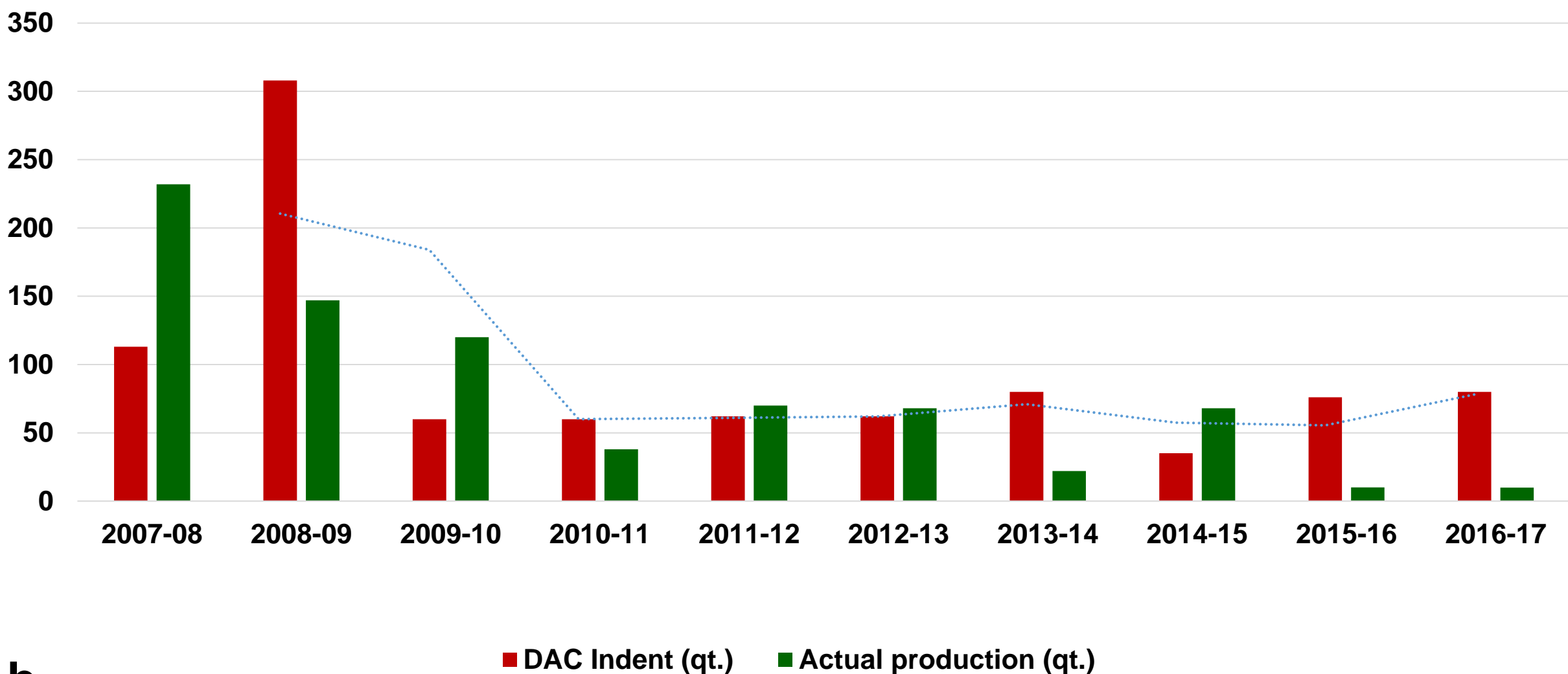

Supplement: Supplementary file 1 — Trends in National indent and actual production of breeders seed of popular varieties (a) Annigeri 1 (b) JG 74 from 2008–2009 to 2016–2017 as per Department of Agriculture and Cooperation, Ministry of Agriculture and Farmers’ Welfare, Government of India (PDF 10 kb) [file 11032_2018_908_MOESM1_ESM.pdf]

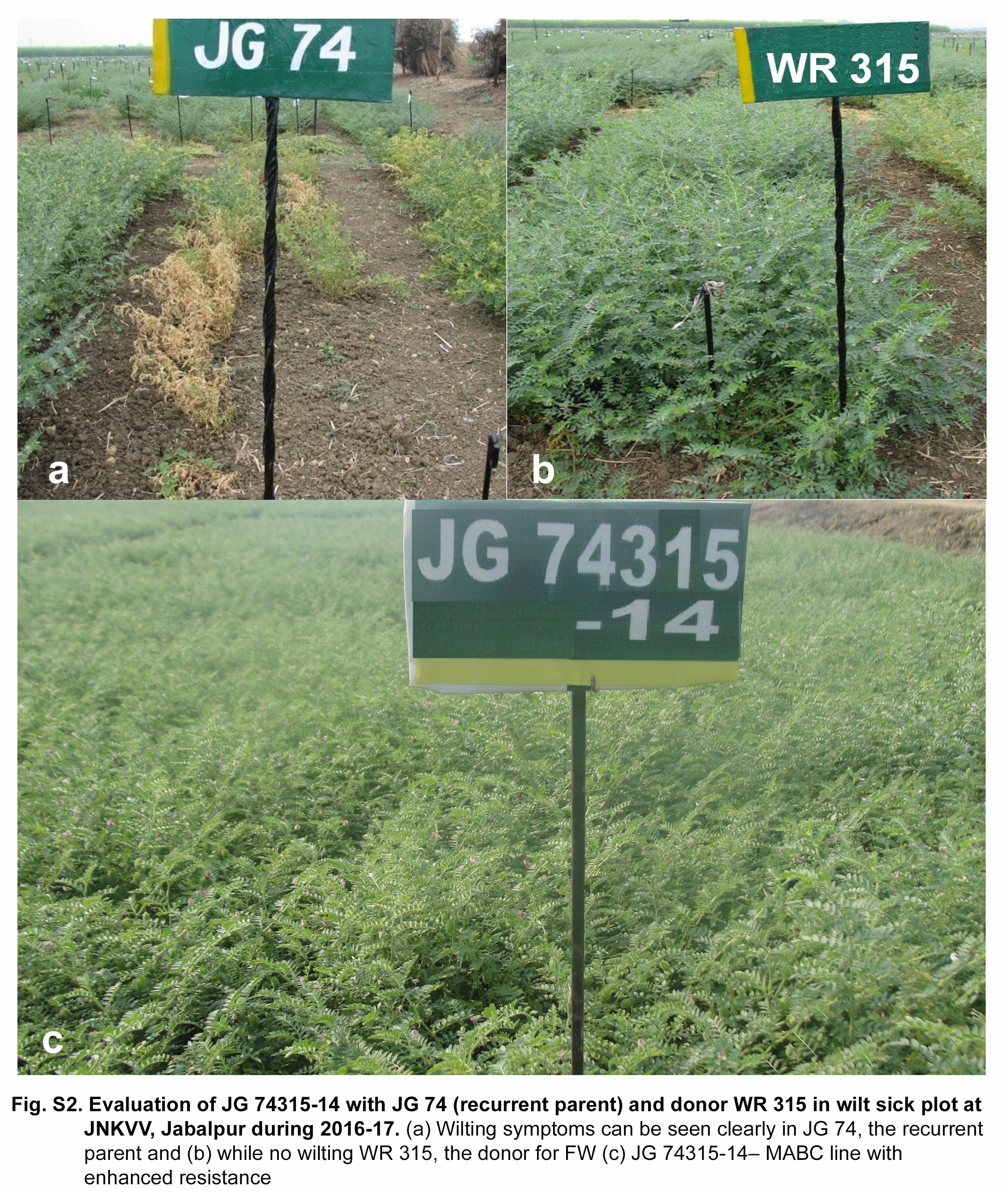

Supplement: Supplementary file 2 — Evaluation of JG 74315-14 with JG 74 (recurrent parent) and donor WR 315 in wilt sick plot at JNKVV, Jabalpur during 2016–2017. (a) Wilting symptoms can be seen clearly in JG 74, the recurrent parent and (b) while no wilting WR 315, the donor for FW (c) JG 74315-14–MABC line with enhanced resistance (JPG 4545 kb) [file 11032_2018_908_MOESM2_ESM.jpg]
