# Supplementary material for: Super Annigeri 1 and improved JG 74: two Fusarium wilt-resistant introgression lines developed using marker-assisted backcrossing approach in chickpea (Cicer arietinum L.)
Source: Mol Breed. 2018 Dec 28;39(1):2. doi: 10.1007/s11032-018-0908-9 (PMC6308216; doi:10.1007/s11032-018-0908-9)
Supplement: Supplementary file 4 — Genetic position of SSR markers used for foreground and background selection of MABC lines in Annigeri 1 and JG 74 genetic backgrounds (DOCX 17 kb) [file 11032_2018_908_MOESM4_ESM.docx]

**Table S2. Genetic position of markers used for foreground and background selection of MABC lines in Annigeri 1 and JG 74 genetic backgrounds**

| **Marker name** | **Linkage group** | **Position**  **(cM)** | **Foreground and background selection** | |
| --- | --- | --- | --- | --- |
|  |  |  | **Annigeri 1 genetic background** | **JG 74 genetic background** |
| TA8 | CaLG01 | 0 | BC_1_F_1_ | BC_3_F_1_ |
| H1G16 | CaLG01 | 17.8 | BC_1_F_1_ |  |
| TR44 | CaLG01 | 24.4 | BC_2_F_1_ | BC_3_F_1_ |
| CaM0244 | CaLG01 | 56.238 | BC_2_F_1_ | BC_3_F_1_ |
| CaM0594 | CaLG01 | 65.653 | BC_1_F_1_ & BC_2_F_1_ |  |
| CaM0403 | CaLG01 | 70.633 |  | BC_3_F_1_ |
| ICCM0297 | CaLG01 | 77.937 | BC_2_F_1_ |  |
| CaM1402 | CaLG01 | 78.434 |  | BC_3_F_1_ |
| TA176 | CaLG01 | 91.236 | BC_2_F_1_ | BC_3_F_1_ |
| GA16 | CaLG02 | 0 | BC_1_F_1_ & BC_2_F_1_ | Foreground selection |
| TA194 | CaLG02 | 11.9 | BC_1_F_1_ & BC_2_F_1_ | BC_3_F_1_ |
| TS82 | CaLG02 | 15.9 | Foreground selection |  |
| TA96 | CaLG02 | 27.3 | Foreground selection | Foreground selection |
| TR19 | CaLG02 | 30.6 | Foreground selection |  |
| TA27 | CaLG02 | 34.6 | BC_1_F_1_ & BC_2_F_1_ | BC_3_F_1_ |
| TA37 | CaLG02 | 44.1 | BC_1_F_1_ & BC_2_F_1_ |  |
| H1F05 | CaLG02 | 52.07 | BC_1_F_1_ & BC_2_F_1_ | BC_3_F_1_ |
| CaM1158 | CaLG02 | 60.76 | BC_1_F_1_ & BC_2_F_1_ | BC_3_F_1_ |
| TA72 | CaLG02 | 73.06 | BC_1_F_1_ & BC_2_F_1_ | BC_3_F_1_ |
| TA46 | CaLG02 | 89.686 | BC_1_F_1_ & BC_2_F_1_ | BC_3_F_1_ |
| CaM1515 | CaLG03 | 0 | BC_1_F_1_ & BC_2_F_1_ | BC_3_F_1_ |
| TA34 | CaLG03 | 12.712 | BC_2_F_1_ | BC_3_F_1_ |
| CaM1358 | CaLG03 | 18.79 | BC_1_F_1_ | BC_3_F_1_ |
| CaM0658 | CaLG03 | 30.69 | BC_1_F_1_ |  |
| H1F14 | CaLG03 | 30.82 |  | BC_3_F_1_ |
| CaM0475 | CaLG03 | 31.26 | BC_1_F_1_ |  |
| TA64 | CaLG03 | 51.29 | BC_1_F_1_ | BC_3_F_1_ |
| ICCM0120b | CaLG03 | 60.434 | BC_2_F_1_ | BC_3_F_1_ |
| TA76 | CaLG03 | 85.45 | BC_1_F_1_ |  |
| TS43 | CaLG03 | 86.958 |  | BC_3_F_1_ |
| TA71 | CaLG03 | 87.64 | BC_2_F_1_ |  |
| TR20 | CaLG04 | 0 | BC_1_F_1_ & BC_2_F_1_ | BC_3_F_1_ |
| H1B17 | CaLG04 | 13.6 | BC_2_F_1_ |  |
| TS72 | CaLG04 | 23.2 | BC_1_F_1_ | BC_3_F_1_ |
| CaM1451 | CaLG04 | 48.261 |  | BC_3_F_1_ |
| CaM1502 | CaLG04 | 57.02 | BC_1_F_1_ |  |
| ICCM0127 | CaLG04 | 58.84 |  | BC_3_F_1_ |
| H1H13 | CaLG04 | 60.26 | BC_1_F_1_ | BC_3_F_1_ |
| CaM0436 | CaLG04 | 63.63 | BC_1_F_1_ |  |
| TA2 | CaLG04 | 72.39 | BC_1_F_1_ | BC_3_F_1_ |
| CaM0038 | CaLG05 | 0 | BC_1_F_1_ & BC_2_F_1_ | BC_3_F_1_ |
| CaM0639 | CaLG05 | 14.728 | BC_2_F_1_ |  |
| TA5 | CaLG05 | 16.389 | BC_2_F_1_ |  |
| H1E22 | CaLG05 | 29.235 | BC_2_F_1_ | BC_3_F_1_ |
| CaM0881 | CaLG05 | 36.93 | BC_1_F_1_ |  |
| TS19 | CaLG05 | 49.031 | BC_2_F_1_ | BC_3_F_1_ |
| CaM0463 | CaLG05 | 61.02 | BC_1_F_1_ & BC_2_F_1_ | BC_3_F_1_ |
| CaM0805 | CaLG05 | 81.49 | BC_2_F_1_ | BC_3_F_1_ |
| ICCM0076 | CaLG05 | 85.94 | BC_1_F_1_ |  |
| H1I16 | CaLG06 | 0 | BC_2_F_1_ | BC_3_F_1_ |
| H1N12 | CaLG06 | 7.876 | BC_2_F_1_ |  |
| CaM0421 | CaLG06 | 28.519 | BC_1_F_1_ | BC_3_F_1_ |
| ICCM0034 | CaLG06 | 37.752 | BC_1_F_1_ & BC_2_F_1_ |  |
| GA34 | CaLG06 | 63.89 | BC_1_F_1_ | BC_3_F_1_ |
| CaM0464 | CaLG06 | 70.1 | BC_1_F_1_ |  |
| CaM0620 | CaLG06 | 78.32 | BC_1_F_1_ | BC_3_F_1_ |
| TA106 | CaLG06 | 91.979 |  | BC_3_F_1_ |
| CaM1101 | CaLG06 | 92.63 | BC_2_F_1_ |  |
| H1O12 | CaLG07 | 0 | BC_1_F_1_ & BC_2_F_1_ | BC_3_F_1_ |
| H1C22 | CaLG07 | 24.2 | BC_1_F_1_ | BC_3_F_1_ |
| CaM0443 | CaLG07 | 36.706 | BC_2_F_1_ |  |
| TA78 | CaLG07 | 39.28 |  | BC_3_F_1_ |
| TA180 | CaLG07 | 40.276 | BC_1_F_1_ |  |
| TAA58 | CaLG07 | 45.59 |  | BC_3_F_1_ |
| TA28 | CaLG07 | 46.32 | BC_1_F_1_ |  |
| ICCM0130a | CaLG08 | 0 | BC_2_F_1_ | BC_3_F_1_ |
| CaM0787 | CaLG08 | 12.4 | BC_2_F_1_ |  |
| TS45 | CaLG08 | 21.2 | BC_1_F_1_ | BC_3_F_1_ |
| H5B04 | CaLG08 | 35.555 | BC_1_F_1_ |  |
| TA3 | CaLG08 | 39.233 | BC_2_F_1_ | BC_3_F_1_ |
