# Supplementary material for: Super Annigeri 1 and improved JG 74: two Fusarium wilt-resistant introgression lines developed using marker-assisted backcrossing approach in chickpea (Cicer arietinum L.)
Source: Mol Breed. 2018 Dec 28;39(1):2. doi: 10.1007/s11032-018-0908-9 (PMC6308216; doi:10.1007/s11032-018-0908-9)
Supplement: Supplementary file 5 — Summary of MABC activities for introgressing resistance to race 4 (foc4) into Annigeri 1 variety at ARS-Kalaburagi (DOCX 14 kb) [file 11032_2018_908_MOESM5_ESM.docx]

**Table S3.** Summary of MABC activities for introgressing resistance to race 4 (*foc* 4) into Annigeri 1 variety at ARS-Kalaburagi

| Linkage group | | QTL targeted | | Markers | BC_1_F_1_  plants |  |  | BC_2_F_1_  plants |  |  |
| --- | --- | --- | --- | --- | --- | --- | --- | --- | --- | --- |
| Foreground selection | | | | | Analyzed | Scorable bands | Heterozygotes | Analyzed | Scorable bands | Heterozygotes |
| CaLG-02 | *foc* 4 | | TA96 | | 188 | 177 | 94 | 376 | 358 | 146 |
| CaLG-02 | *foc* 4 | | TS82 | | 188 | 183 | 96 | 376 | 267 | 79 |
| CaLG-02 | *foc* 4 | | TR19 | | 188 | 46 | 45 | 376 | 357 | 153 |
| Heterozygotes in case of BC_1_F_1_ & BC_2_F_1_ for undertaking background selection | | | | | | | 42 |  |  | 67 |
| Number of SSR markers used for background selection | | | | | | | 38 |  |  | 35 |
| Number of plants after background selection (with % recurrent parent genome recovery) ^†^ | | | | | |  | 42  (58-87 %) |  |  | 67  (78-94%) |
| Number of plants selected with higher background genome recovery used for next backcrossing | | | | | |  | 14  (76-87 %) |  |  | 18  (90-95%) |

^†^ % genome is based on BC_2_F_1_ generation and pedigree
