# Supplementary material for: Super Annigeri 1 and improved JG 74: two Fusarium wilt-resistant introgression lines developed using marker-assisted backcrossing approach in chickpea (Cicer arietinum L.)
Source: Mol Breed. 2018 Dec 28;39(1):2. doi: 10.1007/s11032-018-0908-9 (PMC6308216; doi:10.1007/s11032-018-0908-9)
Supplement: Supplementary file 6 — Percent recurrent parent genome recovery in BC2F1 progenies of cross Annigeri 1 × WR 315 (DOCX 14 kb) [file 11032_2018_908_MOESM6_ESM.docx]

**Table S4.** Percent recurrent parent genome recovery in BC_2_F_1_ progenies of cross Annigeri 1 × WR 315

| **S. No** | **BC_2_F_1_ plant number** | **Recurrent parent genome recovery (%)** |
| --- | --- | --- |
| 1 | 75-167 | **95** |
| 2 | 155-40 | **93** |
| 3 | 61-108 | **93** |
| 4 | 151-99 | **92** |
| 5 | 57-125 | **92** |
| 6 | 50-321 | **91** |
| 7 | 148-212 | **91** |
| 8 | 155-3 | **91** |
| 9 | 155-255 | **91** |
| 10 | 155-52 | **91** |
| 11 | 155-6 | 90 |
| 12 | 75-160 | 90 |
| 13 | 155-9 | 90 |
| 14 | 61-336 | 90 |
| 15 | 50-310 | 90 |
| 16 | 57-361 | 90 |
| 17 | 155-66 | 90 |
| 18 | 155-281 | 90 |
