# Supplementary material for: Super Annigeri 1 and improved JG 74: two Fusarium wilt-resistant introgression lines developed using marker-assisted backcrossing approach in chickpea (Cicer arietinum L.)
Source: Mol Breed. 2018 Dec 28;39(1):2. doi: 10.1007/s11032-018-0908-9 (PMC6308216; doi:10.1007/s11032-018-0908-9)
Supplement: Supplementary file 7 — Details of BC2F3 seeds harvested from at ARS-Kalaburagi during crop season 2013–14 (DOCX 14 kb) [file 11032_2018_908_MOESM7_ESM.docx]

**Table S5.** Details of BC_2_F_3_ seeds harvested from wilt sick screening plot at ARS-Kalaburagi during crop season 2013-14

| **Entry name** | **Total number**  **of plants** | **Number of**  **wilted**  **plants** | **Number of**  **resistant**  **plants** | **BC_2_F_3_ seeds obtained** |
| --- | --- | --- | --- | --- |
| RWRB -50-310-90 | 64 | 23 | 41 | 3,873 |
| RWRB -50-321-91 | 39 | 36 | 3 | 168 |
| RWRB -57-361-90 | 129 | 116 | 13 | 1,008 |
| RWRB -61-336-90 | 161 | 161 | 0 | 0 |
| RWRB -75-160-90 | 6 | 5 | 1 | 54 |
| RWRB -148-212-91 | 35 | 35 | 0 | 0 |
| RWRB -155-6-90 | 8 | 6 | 2 | 132 |
| RWRB -155-9-90 | 29 | 17 | 12 | 745 |
| RWRB -155-40-93 | 1 | 1 | 0 | 0 |
| RWRB -155-52-90 | 11 | 11 | 0 | 0 |
| RWRB -155-66-90 | 146 | 126 | 20 | 1423 |
| RWRB -155-225-91 | 61 | 52 | 9 | 823 |
| RWRB -155-281-90 | 245 | 227 | 18 | 931 |
| **Total** | **935** | **816** | **119** | **9,157** |
