# Supplementary material for: Super Annigeri 1 and improved JG 74: two Fusarium wilt-resistant introgression lines developed using marker-assisted backcrossing approach in chickpea (Cicer arietinum L.)
Source: Mol Breed. 2018 Dec 28;39(1):2. doi: 10.1007/s11032-018-0908-9 (PMC6308216; doi:10.1007/s11032-018-0908-9)
Supplement: Supplementary file 8 — Summary of MABC activities for introgressing resistance to race 4 (foc4) in JG 74 variety using JG 74 × WR 315 cross at JNKVV, Jabalpur (DOCX 14 kb) [file 11032_2018_908_MOESM8_ESM.docx]

**Table S6.** Summary of MABC activities for introgressing resistance to race 4 (*foc 4*) in JG 74 variety using JG 74 × WR 315 cross at JNKVV, Jabalpur

| Linkage group | Gene targeted | Targeted markers | BC_1_F_1_  plants | | | BC_2_F_1_  plants | | | BC_3_F_1_  plants |  |  |
| --- | --- | --- | --- | --- | --- | --- | --- | --- | --- | --- | --- |
| Foreground selection |  |  | Analyzed | Scorable | Heterozygotes | Analyzed | Scorable | Heterozygotes | Analyzed | Scorable | Heterozygotes |
| LG-02 | *foc 4* | TA96 | 6 | 3 | 3 | 21 | - | 4 | 15 | 15 | 15 |
| LG-02 | *foc 4* | GA16 | 6 | 3 | 3 | 21 | - | 4 | 15 | 15 | 15 |
| Common heterozygotes in case of BC_3_F_1_ for both markers  for background selection |  |  |  |  |  |  |  |  |  |  | 15 |
| Number of SSR markers used for background selection |  |  |  |  |  |  |  |  |  |  | 42 |
| Number of plants after background selection  (with % recurrent parent genome recovery) |  |  |  |  |  |  |  |  |  |  | 15  (52-97%) |
| Number of plants selected with higher background genome recovery |  |  |  |  |  |  |  |  |  |  | 10  (91-95%) |
