# Supplementary material for: Super Annigeri 1 and improved JG 74: two Fusarium wilt-resistant introgression lines developed using marker-assisted backcrossing approach in chickpea (Cicer arietinum L.)
Source: Mol Breed. 2018 Dec 28;39(1):2. doi: 10.1007/s11032-018-0908-9 (PMC6308216; doi:10.1007/s11032-018-0908-9)
Supplement: Supplementary file 9 — Details of BC3F2 seeds in the genetic background of JG 74 and germination status in the field (DOCX 15 kb) [file 11032_2018_908_MOESM9_ESM.docx]

**Table S7.** Details of BC_3_F_2_ seeds in the genetic background of JG 74 and germination status in the field

| **BC_3_F_1_ plant number** | **Recurrent**  **parent genome recovery**  **(% )** | **Number of BC_3_F_2_seeds harvested** | **Number of plants emerged (BC_3_F_2_)** | **Number of plants selected based on JG 74 plant type** | **Number of seeds (BC_3_F_3_) harvested** |
| --- | --- | --- | --- | --- | --- |
| 1 | 97 | 168 | 99 | 42 | 6,857 |
| 2 | 96 | 170 | 116 | 47 | 5,999 |
| 3 | 65 | 88 | 50 | - | - |
| 4 | 58 | 236 | 105 | * | * |
| 5 | 93 | 94 | 65 | 41 | 6,239 |
| 6 | 52 | 186 | 115 | - | - |
| 7 | 68 | 5 | - | - | - |
| 8 | 92 | 200 | 124 | 44 | 7,296 |
| 9 | 65 | 6 | - | - | - |
| 10 | 94 | 201 | 132 | 42 | 7,323 |
| 11 | 93 | 278 | 128 | 47 | 8,834 |
| 12 | 96 | 97 | 82 | 45 | 7,235 |
| 13 | 96 | 154 | 102 | 27 | 4,863 |
| 14 | 95 | 43 | 35 | 21 | 3,507 |
| 15 | 90 | 193 | 100 | 38 | 7,923 |
| **Total** |  | **2,119** | **1,253** | **394** | **66,076** |
