# Supplementary material for: Super Annigeri 1 and improved JG 74: two Fusarium wilt-resistant introgression lines developed using marker-assisted backcrossing approach in chickpea (Cicer arietinum L.)
Source: Mol Breed. 2018 Dec 28;39(1):2. doi: 10.1007/s11032-018-0908-9 (PMC6308216; doi:10.1007/s11032-018-0908-9)
Supplement: Supplementary file 10 — Yield performance and disease reaction of 10 best lines in wilt sick plot at ARS-Kalaburagi during 2015–2016 (DOCX 14 kb) [file 11032_2018_908_MOESM10_ESM.docx]

**Table S8.** Yield performance and disease reaction of 10 best lines in wilt sick plot at ARS-Kalaburagi during 2015-16

| **Entry names** | **Yield (kg/ha)** | **Wilt incidence (%)** | **Disease reaction** |
| --- | --- | --- | --- |
| SA1-1 | 1708.33 | 0.00 | R |
| SA1-2 | 1485.42 | 0.00 | R |
| SA1-3 | 1366.67 | 0.00 | R |
| SA1-4 | 1525.00 | 1.35 | R |
| SA1-5 | 1677.08 | 1.52 | R |
| SA1-6 | 1370.83 | 1.85 | R |
| SA1-7 | 1504.17 | 2.38 | R |
| SA18 | 1518.75 | 3.03 | R |
| SA1-9 | 1795.83 | 3.33 | R |
| SA1-10 | 1894.79 | 4.35 | R |
| Annigeri 1 (Recipient) | 1575.00 | 70.00 | S |
| WR 315 (Donor) | 1685.00 | 0.00 | R |
| JG 62 (Susceptible check) | - | 100.0 | HS |
| CD (0.05) | 379.28 |  |  |
| CV (%) | 12.93 |  |  |
