# Supplementary material for: Super Annigeri 1 and improved JG 74: two Fusarium wilt-resistant introgression lines developed using marker-assisted backcrossing approach in chickpea (Cicer arietinum L.)
Source: Mol Breed. 2018 Dec 28;39(1):2. doi: 10.1007/s11032-018-0908-9 (PMC6308216; doi:10.1007/s11032-018-0908-9)
Supplement: Supplementary file 11 — Analysis of variance for yield performance of Super Annigeri lines during 2016–2017 (DOCX 12 kb) [file 11032_2018_908_MOESM11_ESM.docx]

**Table S9. Analysis of variance for yield performance of Super Annigeri lines during 2016-17 at ARS Kalaburagi**

| **Source of variation** | **Degrees of freedom** | **Pooled** | **Bidar** | **Dharwad** | **Kalaburagi** |
| --- | --- | --- | --- | --- | --- |
| Location | 2 | 511.08^**^ | - | - | - |
| Genotype | 12 | 5.01^**^ | 2.71^*^ | 2.20^*^ | 8.16^**^ |
| Location × Genotype | 24 | 4.80^**^ | - | - | - |

^*^Significant at p value <0.05; ^**^ significant at p value <0.01
