# Supplementary material for: Super Annigeri 1 and improved JG 74: two Fusarium wilt-resistant introgression lines developed using marker-assisted backcrossing approach in chickpea (Cicer arietinum L.)
Source: Mol Breed. 2018 Dec 28;39(1):2. doi: 10.1007/s11032-018-0908-9 (PMC6308216; doi:10.1007/s11032-018-0908-9)
Supplement: Supplementary file 12 — Yield performance of Super Annigeri line (MLT-SA-1) over recurrent parent, donor and local check during 2016–2017 (DOCX 12 kb) [file 11032_2018_908_MOESM12_ESM.docx]

**Table S10. Yield performance of Super Annigeri line (MLT-SA-1) over recurrent parent, donor and local check during 2016-17 at ARS Kala**

| **Genotype** | **Pooled** | **Bidar** | **Dharwad** | **Kalaburagi** |
| --- | --- | --- | --- | --- |
| Annigeri 1 (Recurrent parent) | 1786.09 | 1488.42^**^ | 1186.09 | 2683.75 |
| JG 11 (Local check) | 1676.52^**^ | 1738.43 | 1176.56 | 2114.58^**^ |
| WR 315 (Donor parent) | 1572.94^**^ | 1430.55^**^ | 1347.74 | 1940.52^**^ |
| MLT-SA1-1 | 1939.68 | 1840.55 | 1209.74 | 2768.75 |

^**^ significant at p value <0.01
