# Supplementary material for: Super Annigeri 1 and improved JG 74: two Fusarium wilt-resistant introgression lines developed using marker-assisted backcrossing approach in chickpea (Cicer arietinum L.)
Source: Mol Breed. 2018 Dec 28;39(1):2. doi: 10.1007/s11032-018-0908-9 (PMC6308216; doi:10.1007/s11032-018-0908-9)
Supplement: Supplementary file 14 — ANOVA for yield performance of JG 74315-14 at four locations of Madhya Pradesh in state varietal trial during 2016–2017 (DOCX 12 kb) [file 11032_2018_908_MOESM14_ESM.docx]

**Table S12. ANOVA for yield performance of JG 74315-14 at four locations of Madhya Pradesh in state varietal trial during 2016-17**

| **Source of Variation** | **Degrees of freedom** | **Pooled** | **Ganjbasoda** | **Jabalpur** | **Rewa** | **Sagar** |
| --- | --- | --- | --- | --- | --- | --- |
| Location | 3 | 15.15^**^ | - | - | - | - |
| Genotype | 12 | 67.49^**^ | 20.70^**^ | 18.34^**^ | 14.32^**^ | 22.26^**^ |
| Location × Genotype | 36 | 2.83^**^ | - | - | - | - |

^**^ significant at p value <0.01
