# Supplementary material for: Super Annigeri 1 and improved JG 74: two Fusarium wilt-resistant introgression lines developed using marker-assisted backcrossing approach in chickpea (Cicer arietinum L.)
Source: Mol Breed. 2018 Dec 28;39(1):2. doi: 10.1007/s11032-018-0908-9 (PMC6308216; doi:10.1007/s11032-018-0908-9)
Supplement: Supplementary file 15 — Analysis of variance for yield performance of JG 74315-14 over recurrent parent and local check during 2016–2017 (DOCX 12 kb) [file 11032_2018_908_MOESM15_ESM.docx]

**Table S13. Yield performance of JG 74315-14 over recurrent parent and local check during 2016-17**

| **Genotype** | **Pooled** | **Ganjbasoda** | **Jabalpur** | **Rewa** | **Sagar** |
| --- | --- | --- | --- | --- | --- |
| JG 14 (Local check) | 2038.68^**^ | 2006.6^**^ | 2286.73^*^ | 1750.3^**^ | 2111.1^**^ |
| JG 74 (Recurrent parent) | 1128.26^**^ | 1169.22^**^ | 1130.4^**^ | 1258.17^**^ | 955.23^**^ |
| JG 74315-2010-14 | 2549.68 | 2523.24 | 2631.87 | 2399.2 | 2644.43 |

^*^Significant at p value <0.05; ^**^ significant at p value <0.01
