# Supplementary material for: Super Annigeri 1 and improved JG 74: two Fusarium wilt-resistant introgression lines developed using marker-assisted backcrossing approach in chickpea (Cicer arietinum L.)
Source: Mol Breed. 2018 Dec 28;39(1):2. doi: 10.1007/s11032-018-0908-9 (PMC6308216; doi:10.1007/s11032-018-0908-9)
Supplement: Supplementary file 16 — Supplementary Information 1 Yield performance of JG 74315-14, a superior line in the genetic background of JG 74 in IVT trials during 2016–2017 (DOCX 763 kb) [file 11032_2018_908_MOESM16_ESM.docx]

**Supplementary Information 1:** Yield performance of JG 74315-14, a superior line in the genetic background of JG 74 in IVT trials during 2016-17

**
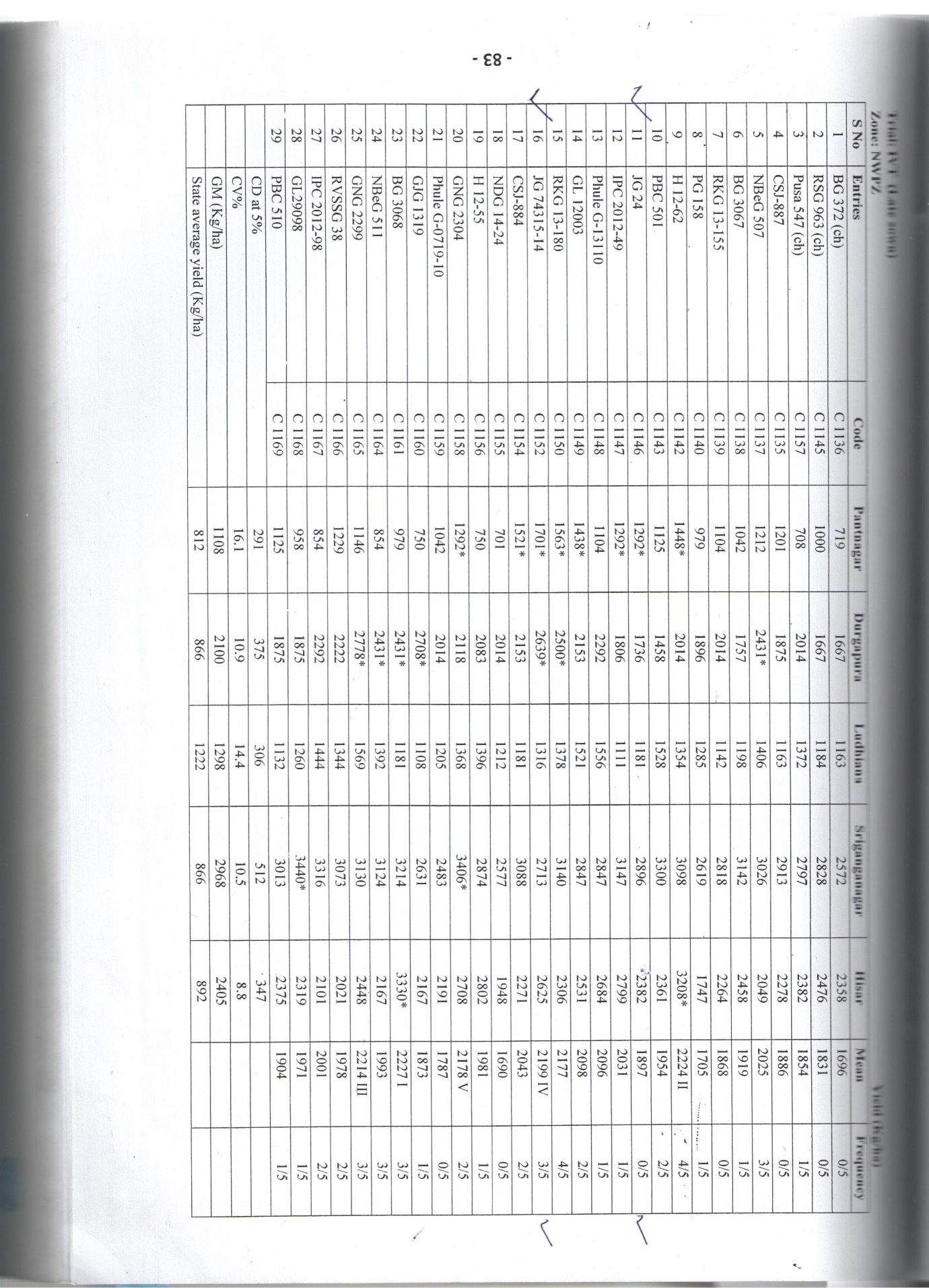
**
